# Improving the Chromosome-Level Genome Assembly of the Siamese Fighting Fish (*Betta splendens*) in a University Master’s Course

**DOI:** 10.1534/g3.120.401205

**Published:** 2020-06-05

**Authors:** Stefan Prost, Malte Petersen, Martin Grethlein, Sarah Joy Hahn, Nina Kuschik-Maczollek, Martyna Ewa Olesiuk, Jan-Olaf Reschke, Tamara Elke Schmey, Caroline Zimmer, Deepak K. Gupta, Tilman Schell, Raphael Coimbra, Jordi De Raad, Fritjof Lammers, Sven Winter, Axel Janke

**Affiliations:** * LOEWE-Centre for Translational Biodiversity Genomics, Senckenberg Nature Research Society, Frankfurt, Germany,; ^†^ South African National Biodiversity Institute, National Zoological Garden, Pretoria, South Africa,; ^‡^ Institute for Ecology, Evolution and Diversity, Goethe University, Frankfurt, Germany, and; ^§^ Senckenberg Biodiversity and Climate Research Centre, Frankfurt, Germany

**Keywords:** chromosome-level genome assembly, *Betta splendens*, master’s course

## Abstract

Ever decreasing costs along with advances in sequencing and library preparation technologies enable even small research groups to generate chromosome-level assemblies today. Here we report the generation of an improved chromosome-level assembly for the Siamese fighting fish (*Betta splendens*) that was carried out during a practical university master’s course. The Siamese fighting fish is a popular aquarium fish and an emerging model species for research on aggressive behavior. We updated the current genome assembly by generating a new long-read nanopore-based assembly with subsequent scaffolding to chromosome-level using previously published Hi-C data. The use of ∼35x nanopore-based long-read data sequenced on a MinION platform (Oxford Nanopore Technologies) allowed us to generate a baseline assembly of only 1,276 contigs with a contig N50 of 2.1 Mbp, and a total length of 441 Mbp. Scaffolding using the Hi-C data resulted in 109 scaffolds with a scaffold N50 of 20.7 Mbp. More than 99% of the assembly is comprised in 21 scaffolds. The assembly showed the presence of 96.1% complete BUSCO genes from the Actinopterygii dataset indicating a high quality of the assembly. We present an improved full chromosome-level assembly of the Siamese fighting fish generated during a university master’s course. The use of ∼35× long-read nanopore data drastically improved the baseline assembly in terms of continuity. We show that relatively in-expensive high-throughput sequencing technologies such as the long-read MinION sequencing platform can be used in educational settings allowing the students to gain practical skills in modern genomics and generate high quality results that benefit downstream research projects.

The Siamese fighting fish, *Betta splendens*, is known for its eponymic aggressive behavior between conspecific males. It was introduced into the international aquarium trade from the wild almost 130 years ago. The wildtype of *B. splendens* is endemic to Thailand and inhabits intact marshlands in shallow zones (Vidthayanon 2012). It is classified as “vulnerable” by the International Union for Conservation of Nature (IUCN) with decreasing population trends due to habitat destruction and pollution (Vidthayanon 2012). As a popular aquarium fish, it has been under strong artificial selection to produce several morphotypical variants as well as heightened aggressive behavior. Numerous studies have focused on the psychological (Eisenreich *et al.*, 2017), behavioral (Dzieweczynski and Kane 2017) and ecological aspects (Castro *et al.*, 2006) of this artificial selection. Genetic studies mostly investigated the genetic basis of the manifold of colors and fin shapes found in this species (Goodrich and Mercer 1934).

Recently, Fan *et al.* (2018) generated a chromosome-level *B. splendens* reference assembly. In order to do so, they first generated a baseline assembly using a combination of paired-end and mate pair libraries (sequenced on the Illumina HiSeq2000 platform), and then super-scaffolded the resulting assembly using a proximity-ligation-based Hi-C library (sequenced on the BGISEQ-500 platform). To further improve this assembly and to provide a solid basis for future analyses on this important fish model, we generated a more continuous baseline assembly using long-read data generated with the MinION sequencing device from Oxford Nanopore Technologies (ONT), and subsequently carried out scaffolding using the published Hi-C data from Fan *et al.* (2018).

Data generation and genome assembly was performed by students in the framework of a six-week master’s course. This demonstrates the great potential of newly developed genome sequencing technologies for education. We hope that our study encourages academic institutions to offer hands-on genomics courses to students to gain first-hand experience in working with genomic data.

## Materials and Methods

### DNA extraction and sequencing

We extracted high molecular weight DNA from muscle tissue of two female individuals of aquarium-kept Siamese fighting fish using the protocol described in Mayjonade *et al.*, (2016). Two individuals were used due to their small size and the fact that the muscle tissue of one individual did not yield sufficient high molecular weight DNA (hmwDNA) for all sequencing runs. Aquarium fish that are bred in captivity, such as *Betta splendens*, are usually very inbred, which reduces the variation between individuals to a minimum. DNA quantity and fragment lengths were checked using the Genomic DNA ScreenTape (TapeStation Analysis Software A.02.01 SR1). We prepared four sequencing libraries using ONT’s Rapid (SQK-RAD004; three libraries) and 1D (SQK–LSK109; one library) sequencing kits. The resulting libraries were sequenced on individual R9.4 flow cells using a ONT MinION.

### Genome assembly and scaffolding

We used Albacore v.2.3.3 (https://community.nanoporetech.com) for base-calling of the raw reads and removed reads with average quality scores below 7. In order to generate an overlap-layout graph for subsequent assembly, we first used Minimap2 v.2.14-r883 (Li 2018) to carry out all-*vs.*-all mapping using the default parameters for ONT data. Subsequently, we used Miniasm v.0.3-r179 (Li 2016) to generate the assembly graph and converted the resulting gfa file into a consensus sequence fasta file using awk (Unix scripting language). For consensus polishing, we first aligned the nanopore reads back to our assembly using Minimap2 and performed the error correction using Racon v.1.3.1 (Vaser *et al.*, 2017). This step was repeated twice. Next, to further improve the resulting consensus quality, we performed error correction using previously published Illumina paired-end short-read data (accession no. SRR6251365; Fan *et al.* (2018)). For that, we first used Cutadapt v.1.18 (Martin 2011) using default values to remove adapter sequences as well as low-quality ends from the reads. We then mapped the paired-end (SRR6251365) and mate pair (SRR6251353) data onto the genome assembly using BWA-MEM v.0.7.17-r1188 (Li and Durbin 2010) and sorted the resulting mapping file using SAMtools v.1.9 (Li *et al.*, 2009). Lastly, we ran three rounds of the polishing using Pilon v.1.23 (Walker *et al.*, 2014).

In order to achieve chromosome-level for our long-read based assembly, we removed all contigs matching to the mitochondrial genome using default values in blast (with a minimum identity of 90% and a minimum lengths of 1 kbs), and subsequently mapped the previously published Hi-C reads (accession no. SRR6251367; (Fan *et al.*, 2018) onto the genome using BWA-MEM. Next, we scaffolded the assembly using the Hi-C reads with ALLHic v.0.9.8 (Zhang *et al.*, 2019) using default values, except for -e GATC and -k 21. We then performed one last round of short-read (SRR6251367) based polishing using Pilon v.1.23 (Walker *et al.*, 2014).

### Genome quality assessment

**Figure 1:**
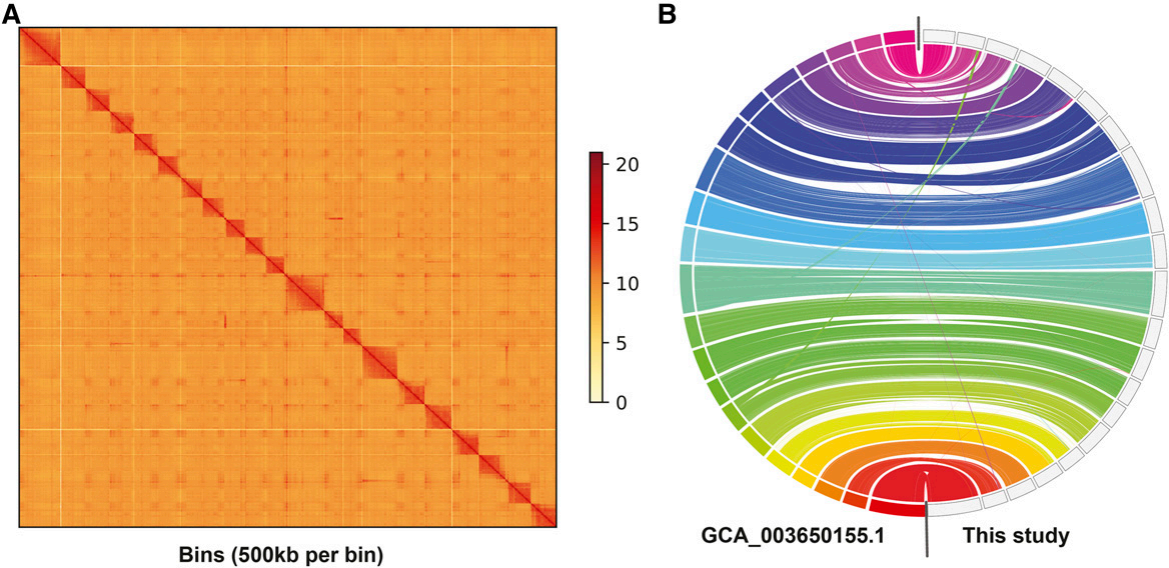
(A) Hi-C contact map of the 21 chromosome-level scaffolds, and the shorter unplaced scaffolds. As can be seen in the plot, the assembly only shows small amounts of *trans*-chromosomal interactions. The scale revers to the link coverage along and between scaffolds. (B) Whole genome synteny between the chromosome-level assembly of Fan *et al.* (2018) (on the left) and our chromosome-level assembly (on the right). The lines indicate aligned regions between the two assemblies.

We obtained genome continuity statistics (Table 1) with QUAST v5.0.2 (Gurevich *et al.*, 2013) and assessed assembly completeness using BUSCO v.4.0.6 (Waterhouse *et al.*, 2018) with the Actinopterygii gene set (actinopterygii_odb10, busco.ezlab.org). We then mapped the Illumina HiSeq2000 reads from the 5 kbp insert-size mate pair library (accession no. SRR625353; Fan *et al.*, (2018)) to the assembly and investigated the distribution of insert sizes for the library (Supplementary Figure 2). We mapped the data using BWA-MEM, sorted the alignment files with SAMtools, marked duplicates using Picard v.2.20.7 (https://broadinstitute.github.io/picard/), and then created a histogram based on the statistics obtained from GATK v.4.1.4.1 (https://gatk.broadinstitute.org; CollectInsertSizeMetrics option). Investigating synteny changes between the two chromosome-level assemblies with JupiterPlot (https://zenodo.org/record/1241235#.XqrE9iOB0pQ), we found a strong overall agreement with some differences especially toward the ends of the scaffolds (Figure 1B). We further investigated the amount of contaminated contigs in our assembly using Blobtools v.1.1.1 (Laetsch and Blaxter 2017).

**Table 1:** Genome continuity statistics for the Hi-C scaffolded genome calculated with QUAST in comparison to the assembly of Fan *et al.* (2018)

	This Study	Fan *et al.* 2018
**Number of contigs/scaffolds**	1,276 /109	139,323/91,819
**Contig N50**	2.1 Mbp	18.9 kbp
**Scaffold N50**	20.7 Mbp	19.7 Mbp
**Scaffold L50**	9	10
**Largest scaffold**	34.1Mbp	34.9 Mbp
**Assembly size**	441.2 Mbp	456.2 Mbp
**GC (%)**	45.1	45.2

### Transcriptome assembly and quality assessment

In order to assemble the transcriptome of *B. splendens* for subsequent use in gene annotation, we downloaded seven previously published RNA-seq libraries from NCBI (accession no. SRR6251368–SRR6251375). We assembled the transcriptomes *de novo* using Oases v.0.2.09 (Schulz *et al.*, 2012). The completeness of the transcriptome assembly was assessed with BUSCO, using the Actinopterygii gene set.

### Genome annotation

#### Repeat annotation:

In order to annotate repeats in our assembly we first created a custom *de novo* repeat library using RepeatModeler v.1.0.11 (www.repeatmasker.org/RepeatModeler/) and then combined this library with the curated *Danio rerio* repeat dataset from Dfam 3.0 (Hubley *et al.*, 2016). Repeats in the genome were then annotated and masked using RepeatMasker open-4.0.7 (www.repeatmasker.org/RepeatMasker/).

#### Gene annotation:

Gene annotation was performed using MAKER2 v.2.31.10 (Holt and Yandell 2011) in several steps. First, we carried out evidence-based annotation using proteins obtained from Fan *et al.* (2018); available at gigadb.org/dataset/100433) and our aforementioned *de novo* assembled transcriptomes. We then trained the *ab initio* gene predictor SNAP v.2006-07-28 (Korf 2004) using MAKER2 results over two rounds. Additionally, we used the two *ab initio* gene predictors Augustus v.3.3 (Stanke *et al.*, 2008) and Genemark v.4.38 (Lomsadze *et al.*, 2005). We applied BUSCO and DOGMA v.3.4 (Dohmen *et al.*, 2016) to assess the completeness of the annotated gene models.

### Data availability

The genome assembly and all read data generated during this project are accessible on GenBank (Bioproject PRJNA592275). Supplemental material available at figshare: https://doi.org/10.25387/g3.12273146.

## Results and Discussion

### Genome assembly and annotation

All four sequencing runs yielded a total of ∼21 Gbp of read data, with an average read length N50 of ∼5.8 kbp, ranging from 1.2 to 8.6 kbp for the different sequencing runs (Supplementary Figure 1 and Supplementary Table 1). After base-calling and filtering we retained 18 Gbp of sequencing reads. Subsequent assembly resulted in a genome size of 441 Mbp with 1,276 contigs and a N50 of 2.1 Mbp, which is a substantial improvement to the 19 kbp contig N50 from Fan *et al.* (2018). Hi-C data based scaffolding resulted in 109 scaffolds with a scaffold N50 of 20.7 Mbp (Table 1). Over 99% of the assembly size was placed into 21 chromosome-length scaffolds, compared to the 95.3% of Fan *et al.* (2018). A contact map of the resulting assembly can be seen in Figure 1A. This map shows only little *trans*-chromosomal interactions in our genome assembly.

Genome quality assessment resulted in the recovery of 96.9% complete BUSCO’s (96.1% single-copy and 0.8% duplicated complete genes) (Table 2). The assembly showed 101 missing genes out of a total of 3,640 BUSCO’s investigated (2.8%). This is comparable to the BUSCO scores obtained from the chromosome-level assembly of Fan *et al.* (2018), with a slightly higher rate of missing BUSCO’s (2.8% compared to 2.4% in Fan *et al.* (2018); see Table 2). We then further investigated the quality of the assembly using mate-pair short-read data.

**Table 2:** Comparison of BUSCO scores for our and the chromosome-level assembly of Fan *et al.* (2018). We did not include complete single-copy and duplicated BUSCO statistics for the transcriptome assembly, as it includes isoforms. The analyses are based on a total number of 3,640 BUSCOs

	Genome		Annotation		Transcriptome
	this study	Fan *et al.* 2018	this study	Fan *et al.* 2018	this study
**Complete BUSCOs (C)**	3,528 (96.9%)	3,543 (97.3%)	2,870 (78.8%)	3,164 (87.0%)	3,194 (87.7%)
**Complete and single-copy BUSCOs (S)**	3,499 (96.1%)	3,498 (96.1%)	2,819 (77.4%)	3,093 (85.0%)	—
**Complete and duplicated BUSCOs (D)**	29 (0.8%)	45 (1.2%)	51 (1.4%)	71 (2.0%)	—
**Fragmented BUSCOs (F)**	11 (0.3%)	11 (0.3%)	148 (4.1%)	225 (6.2%)	149 (4.1%)
**Missing BUSCOs (M)**	101 (2.8%)	86 (2.4%)	622 (17.1%)	251 (6.8%)	297 (8.2%)

We observed a much higher rate of read pairs mapping with the expected orientation and insert size in both our polished Nanopore baseline assembly and our final chromosome-level assembly compared to the chromosome-level assembly of Fan *et al.* (2018) (see Supplementary Figure 2). The Blobtools analysis showed no signs of contamination in our genome assembly, as 99.99% of the assembly were taxonomically assigned as Chordata and the majority of the scaffolds and contigs showed highly similar coverage and GC contents (Supplementary Figure 3). We found very narrow peaks for the distributions of coverage and GC content in the assembly.

Next, we annotated the genome assembly. To do so, we first *de novo* assembled the transcriptome. BUSCO analysis revealed 87.7% complete, 4.1% fragmented, and 8.2% of missing BUSCO’s (Table 2). To be able to improve the gene annotation, we first repeatmasked the genome. The results show that our *Betta splendens* genome assembly consists of 27.8% repeats, with LINEs (7.1%) and simple repeats (5.4%) making up the largest fractions of repeats (Table 3). This is higher than the 15.1% reported in Fan *et al.* (2018). The subsequent gene annotation resulted in 21,535 annotated transcripts, which is slightly lower than the 23,981 gene models generated by Fan *et al.* (2018). Within BUSCO, the Actinopterygii set yielded 78.8% (n = 3,640) complete core orthologs and within DOGMA 83.88% (n = 8,113) of the vertebrate sets conserved domain arrangements (CDAs). This is lower than the 87.0% (BUSCO) and the 89.93% (DOGMA) scores we obtained for the annotation of Fan *et al.* (2018). This could be caused by the imperfection of long-read polishing. However, about 90% of all our gene models showed Annotation Edit Distance (AED) < 0.5, which indicates a high quality of the annotated gene models (Supplementary Figure 4). The AED describes the congruency between evidence alignment and predicted gene between 0 and 1, with 0 indicating perfect agreement (Yandell and Ence 2012).

**Table 3:** Repeat content of the Hi-C scaffolded assembly

Type of element	Number of elements	Length	Percentage of assembly
**SINEs**	12,939	2,732,859	0.62%
**LINEs**	51,088	31,431,107	7.13%
**LTR elements**	24,596	21,574,199	4.89%
**DNA transposons**	54,813	14,902,018	3.37%
**Unclassified**	74,783	23,656,821	5.36%
**Small RNA**	6,578	1,201,687	0.27%
**Satellites**	2,397	1,216,117	0.28%
**Simple repeats**	385,394	24,039,213	5.44%
**Low complexity**	35,821	2,031,477	0.46%
		**Total:**	27.82%

### Educational aspect of the assembly generation

The MinION’s potential as an effective teaching tool was recognized early on and it has been used in classroom settings (Salazar *et al.*, 2020; Zaaijer and Erlich 2016; Zeng and Martin 2017) as well as in the field (Watsa *et al.*, 2020). The presented study illustrates that inexpensive nanopore-based sequencing along with published short-read data, as well as memory and run-time efficient genome assembly tools offer great potential to generate high quality chromosome-level assemblies, even of more complex vertebrate genomes, as part of university courses. A more detailed discussion on the educational side and structure of the course can be found in Prost *et al.*, (2020). In short, the teaching included the basics and practical skills needed for extraction of hmwDNA, library preparation and subsequent sequencing on the MinION device. In contrast, we used previously published Hi-C data for the scaffolding, as generating this kind of data adds substantial complexity to the laboratory part of the course and might therefore be overwhelming for students without prior laboratory training. Freely accessible databases such as the DNA Zoo (https://www.dnazoo.org/) offer a great source for Hi-C data. However, it would also be possible to generate such data prior to the course, in case no published data are available. Before we used the long-read data to assemble a highly continuous baseline assembly, we spent two days teaching students the basics of working with the command-line on a Unix server. During the data processing, we first had the students run the tools on a subset of the data, and then divided them into small groups (2-3 students) to run the same tools on the full data set. This way the students were involved in every step of the data processing and analyses during the course. We used memory and run-time efficient genome assembly tools such as Miniasm and subsequent polishing instead of hybrid-assembly tools, as the run time of the latter would not have allowed us to perform all analyses with the total data during the course.

With the described setting, scientific topics like high-throughput sequencing, the bioinformatics of genome assembly and genome evolution can be taught in a highly applied and engaging way. Furthermore, modern technologies do not only offer the chance for invaluable training using state-of-the-art methods, but also allow students to publish results early on in their career. The ever-decreasing sequencing costs should enable universities, even in low-income areas and countries, to train their students in modern genomics and bioinformatics.
